# Genome-Resolved Metagenomics Extends the Environmental Distribution of the *Verrucomicrobia* Phylum to the Deep Terrestrial Subsurface

**DOI:** 10.1128/mSphere.00613-19

**Published:** 2019-12-18

**Authors:** Sophie L. Nixon, Rebecca A. Daly, Mikayla A. Borton, Lindsey M. Solden, Susan A. Welch, David R. Cole, Paula J. Mouser, Michael J. Wilkins, Kelly C. Wrighton

**Affiliations:** a Department of Earth and Environmental Sciences, University of Manchester, Manchester, United Kingdom; b Soil and Crop Sciences, Colorado State University, Fort Collins, Colorado, USA; c Centre for Microbial Pathogenesis, The Research Institute at Nationwide Children’s Hospital, Columbus, Ohio, USA; d School of Earth Sciences, The Ohio State University, Columbus, Ohio, USA; e College of Engineering and Physical Sciences, University of New Hampshire, Durham, New Hampshire, USA; University of Wisconsin-Madison

**Keywords:** glycoside hydrolases, viruses, shale, hypersaline, hydraulic fracturing

## Abstract

Bacteria of the phylum *Verrucomicrobia* are prevalent and are particularly common in soil and freshwater environments. Their cosmopolitan distribution and reported capacity for polysaccharide degradation suggests members of *Verrucomicrobia* are important contributors to carbon cycling across Earth’s ecosystems. Despite their prevalence, the *Verrucomicrobia* are underrepresented in isolate collections and genome databases; consequently, their ecophysiological roles may not be fully realized. Here, we expand genomic sampling of the *Verrucomicrobia* phylum by describing a novel genus, “*Candidatus* Marcellius,” belonging to the order *Opitutales*. “*Ca.* Marcellius” was recovered from a shale-derived produced fluid metagenome collected 313 days after hydraulic fracturing, the deepest environment from which a member of the *Verrucomicrobia* has been recovered to date. We uncover genomic attributes that may explain the capacity of this organism to inhabit a shale gas well, including the potential for utilization of organic polymers common in hydraulic fracturing fluids, nitrogen fixation, adaptation to high salinities, and adaptive immunity via CRISPR-Cas. To illuminate the phylogenetic and environmental distribution of these metabolic and adaptive traits across the *Verrucomicrobia* phylum, we performed a comparative genomic analysis of 31 publicly available, nearly complete *Verrucomicrobia* genomes. Our genomic findings extend the environmental distribution of the *Verrucomicrobia* 2.3 kilometers into the terrestrial subsurface. Moreover, we reveal traits widely encoded across members of the *Verrucomicrobia*, including the capacity to degrade hemicellulose and to adapt to physical and biological environmental perturbations, thereby contributing to the expansive habitat range reported for this phylum.

**IMPORTANCE** The *Verrucomicrobia* phylum of bacteria is widespread in many different ecosystems; however, its role in microbial communities remains poorly understood. *Verrucomicrobia* are often low-abundance community members, yet previous research suggests they play a major role in organic carbon degradation. While *Verrucomicrobia* remain poorly represented in culture collections, numerous genomes have been reconstructed from metagenomic data sets in recent years. The study of genomes from across the phylum allows for an extensive assessment of their potential ecosystem roles. The significance of this work is (i) the recovery of a novel genus of *Verrucomicrobia* from 2.3 km in the subsurface with the ability to withstand the extreme conditions that characterize this environment, and (ii) the most extensive assessment of ecophysiological traits encoded by *Verrucomicrobia* genomes to date. We show that members of this phylum are specialist organic polymer degraders that can withstand a wider range of environmental conditions than previously thought.

## Abstract (precis)

The *Verrucomicrobia* phylum of bacteria is widespread in many different ecosystems; however, its role in microbial communities remains poorly understood. *Verrucomicrobia* are often low-abundance community members, yet previous research suggests they play a major role in organic carbon degradation. While *Verrucomicrobia* remain poorly represented in culture collections, numerous genomes have been reconstructed from metagenomic data sets in recent years. The study of genomes from across the phylum allows for an extensive assessment of their potential ecosystem roles. The significance of this work is (i) the recovery of a novel genus of *Verrucomicrobia* from 2.3 km in the subsurface with the ability to withstand the extreme conditions that characterize this environment, and (ii) the most extensive assessment of ecophysiological traits encoded by *Verrucomicrobia* genomes to date. We show that members of this phylum are specialist organic polymer degraders that can withstand a wider range of environmental conditions than previously thought.

## INTRODUCTION

The *Verrucomicrobia* are a prevalent phylum of *Bacteria* common to soils and freshwater environments, yet members are underrepresented in isolate collections and genome databases (1, 2). Currently, by 16S rRNA gene phylogeny (3), the phylum is composed of a single class (*Verrucomicrobiae*), which has eleven orders, over half of which lack a cultivated representative. Metagenome assembled genomes (MAGs) from numerous and diverse habitats have begun to fill in missing foliage of the *Verrucomicrobia* portion of the tree of life. Based on current estimates, there are a few more than 30 near-complete (estimated completeness, >75%) MAGs, all of which were recovered in the last 5 years (1, 2, 4, 5).

*Verrucomicrobia* have been isolated from diverse habitats, including soil (6–12), freshwater (12), marine (13–17), geothermal (18–20), and hypersaline (21) systems. Additionally, members of this phylum are distributed in host-associated biomes, with recovery of *Verrucomicrobia* genomes from sugar beet roots (22) and the gastrointestinal tracts of termites (23) and humans (24), as well as extreme environments, like cryoconite holes in glaciers (25), the deep (300-m) subsurface (26), and hypersaline lakes (27–29). Taken together, *Verrucomicrobia* are broadly distributed across a range of habitat types.

Most studies addressing *Verrucomicrobia* ecophysiology have focused on soils (11, 12), where *Verrucomicrobia* can represent more than 30% of bacterial 16S rRNA gene sequences (1). Although their diversity in soils is broad (1), the most abundant members are reported to belong to the previously described class *Spartobacter* (30, 31). This class, now recognized as belonging to order *Chthoniobacterales*, was recently described with the recovery of “*Candidatus* Udaeobacter copiosus” (1). Its metabolic reconstruction indicates specialization for a limited range of carbon substrates, suggesting this organism sacrifices metabolic versatility for efficiency (1).

A growing number of studies are also using MAGs to highlight the biogeochemical roles of *Verrucomicrobia* in freshwater habitats. In these habitats, *Verrucomicrobia* can account for up to 19% of the 16S rRNA gene sequences (2, 4, 5). The recovery of large numbers of *Verrucomicrobia* MAGs from aquatic environments indicates these organisms have a remarkable range in genome size, widely encode nutrient limitation strategies, and have the capacity for polysaccharide degradation (2, 4). He et al. (2) reported 19 *Verrucomicrobia* MAGs from a eutrophic lake and humic bog that were enriched with polysaccharide-degrading genes. Transcriptomic evidence suggested that while these freshwater *Verrucomicrobia* were actively degrading carbohydrates in ice-covered freshwater lakes, they may also utilize a chemolithotrophic metabolism under conditions where organic carbon is limited (5). Three *Verrucomicrobia* MAGs were recently recovered from waters sampled 300 to 450 m deep in granitic bedrock (26). Prior to this, the subsurface extent of *Verrucomicrobia* was 5 m, where three MAGs were recovered from aquifer waters (32).

Here, we use fluids produced as a by-product of shale gas production (hydraulic fracturing) as an opportunity to collect microbial biomass from the poorly sampled, deep terrestrial subsurface. Production of shale gas is achieved by hydraulic fracturing, the high-pressure injection of water and chemical additives into hydrocarbon-rich shales. Previous reports have shown that this perturbation to the subsurface creates a new ecosystem, where introduced microorganisms proliferate more than 2,000 m below the surface (33–35). From a produced fluid sample collected 313 days after hydraulic fracturing, we report the recovery of a MAG belonging to a new genus in the phylum *Verrucomicrobia*. Here, we demonstrate that the produced fluid-derived *Verrucomicrobia* genome encodes traits enabling tolerance to the chemical, physical, and biological stressors in this engineered habitat. Our findings extend the depth and salinity range of the *Verrucomicrobia*, thereby expanding the ecophysiological understanding and known environmental distribution of this biogeochemically relevant and cosmopolitan lineage.

## RESULTS AND DISCUSSION

### The microbial ecosystem of a shale gas well in the Marcellus formation.

**FIG 1:**
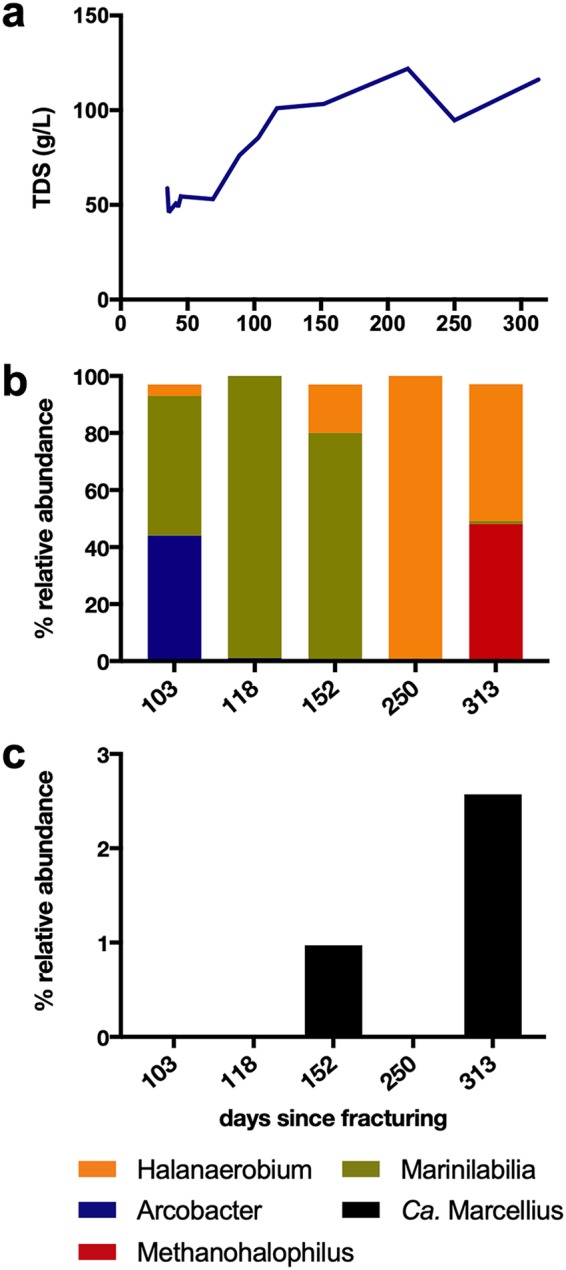
Geochemistry and microbial community attributes of the Marcellus-5 fractured shale ecosystem since the collection of produced fluids on day 34. (a) Produced fluids from 93 days after fracturing onwards are characterized by high salinity, expressed as total dissolved solids (TDS) in grams per liter. (b) Relative abundances of fractured shale members (as inferred from *rpsC* gene relative abundance) across the five metagenomes. (c) “*Candidatus* Marcellius” is a rare member of the community only detected in later metagenomes.

The salinity of produced fluids collected from a Marcellus shale well (Marcellus-5 [33]) range from freshwater levels (upon injection) to a maximum salinity (as total dissolved solids; TDS) of 122 g/liter 215 days after fracturing, with an average of 104 g/liter over the duration of the sampling period (Fig. 1a). This salinity increase is the result of interactions between freshwater-based input fluids and salts in the shale formation (36). Consistent with prior reports by our team and others (34, 35, 37–39), we show that salinity increases in the shale produced fluids over time in the Marcellus-5 well.

To better understand microbial community response to salinity changes, we selected five samples (spanning 103 to 313 days posthydraulic fracturing) where TDS nearly doubled from input fluid concentrations. Microbial relative abundance (based on *rpsC* gene [S3 ribosomal protein] relative abundance) revealed that *Arcobacter* and *Marinilabilia* dominated earlier samples until day 152, with *Methanohalophilus* and *Halanaerobium* dominating later time points (Fig. 1b; see also Table S1 in the supplemental material). These findings are consistent with the recent cultivation of *Arcobacter* and *Methanohalophilus* strains recovered from early and late produced fluids, respectively, from an adjacent shale gas well (40, 41). Moreover, our findings highlight salinity as an environmental filter, likely contributing to the low microbial diversity and enrichment in halotolerant taxa over time.

10.1128/mSphere.00613-19.4 TABLE S1 Abundance of genome bins recovered from the Marcellus-5 shale gas well produced waters based on S3 (*rpsC* gene) abundance. An asterisk indicates the sum of S3 abundances that could be attributed to the taxonomic assignment given in the table. Download Table S1, DOCX file, 0.1 MB. Copyright © 2019 Nixon et al. 2019 Nixon et al. This content is distributed under the terms of the Creative Commons Attribution 4.0 International license.

Notably, we recovered a genome belonging to the *Verrucomicrobia* from the day 313 metagenome (Fig. 1c). Based on *rpsC* gene (S3 ribosomal protein) relative abundance, this genome is a low-abundance member of the community, reaching 2.6% relative abundance 313 days after fracturing. Given this low abundance and that this system was operated in a closed fashion (limited perturbation after initial hydraulic fracturing event), we consider it likely that the genome was not recovered in other samples due to it being below the detection level needed for assembly. Supporting this assumption, mapping reads from the other temporal samples to the day 313 genome demonstrated read recruitment but no assembled contigs from the day 152 metagenome. This organism therefore appears to be a rare member of the fractured shale community (Table S1).

### A novel genus of *Verrucomicrobia* recovered from fractured shale.

From metagenomic data, we reconstructed a nearly complete, high-quality (42) (>99% estimated completeness with <1% contamination based on the presence of single-copy genes (34) from a genome belonging to the *Verrucomicrobia* phylum from the day 313 sample. The recovered genome is 4.10 Mbp in size with an average GC content of 62.3%. It has 3,400 genes coding for predicted proteins on 40 contigs (>5 kb) and includes a 1,425-bp 16S rRNA gene as well as 5S and 23S rRNA genes and 44 tRNA genes. This represents the first nearly complete genome from the *Verrucomicrobia* recovered from hydraulically fractured shales and therefore offers a unique opportunity to understand genome-encoded traits that may extend the depth and salinity range for members of this phylum.

**FIG 2:**
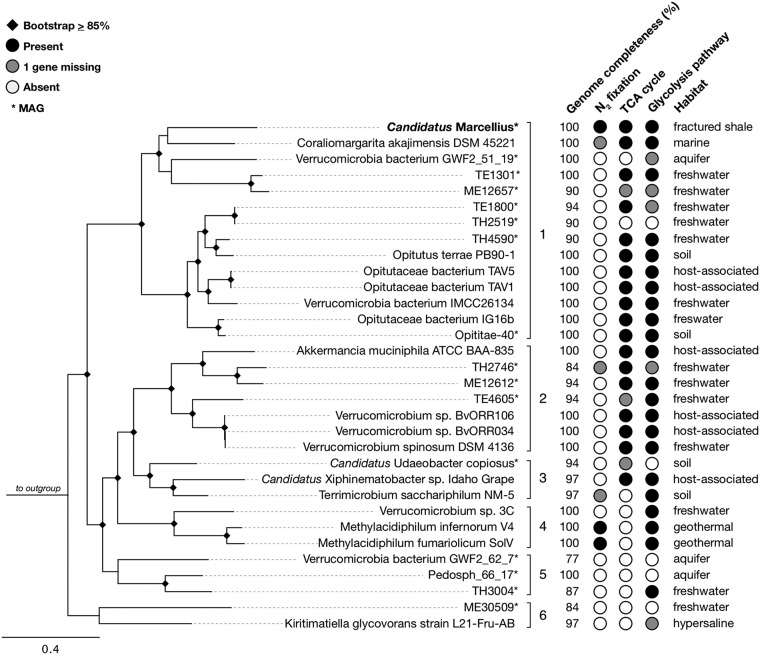
Phylogenetic tree of 32 nearly complete *Verrucomicrobia* genomes, their key attributes, and habitats of origin. “*Ca.* Marcellius” is highlighted in boldface. Metagenome-assembled genomes (MAGs) are denoted with asterisks. Square brackets denote orders containing cultivated representatives. According to Silva (v 132, May 2019), the phylum is comprised of five orders containing cultured representatives: 1, *Optitulates*; 2, *Verrucomicrobiales*; 3, *Chthoniobacterales*; 4, *Methylacidiphilales*; 5, *Pedospherales*. Order 6 represents the formerly acknowledged subdivision 5, the only cultivated member of which (Kiritimatiella glycovorans strain L21-Fru-AB) was recently assigned to the novel sister phylum *Kiritimatiellaeota* (27). The outgroup is Escherichia coli. The tree was constructed from a concatenated alignment of 11 conserved ribosomal genes (amino acid sequences; large subunits L5, L6, L14, L15, L16, L18, L22, and L24 and small subunits S3, S8, and S17) using 100 bootstraps. Bootstrap values equal to or greater than 85% are represented with black diamonds. Genome completeness was estimated from the presence of 31 single-copy genes (34). Genes for the *nifHDKENB* subunits coding for nitrogen fixation, a complete tricarboxylic acid (TCA) cycle, and complete glycolysis pathway were assessed for each genome (present, black circles; absent, white circles; 1 gene missing from complete set, gray circles).

16S rRNA gene and concatenated gene phylogenetic analyses revealed this genome belonged to a new genus within the family *Puniceicoccaceae* of the order *Opitutales* in the *Verrucomicrobia* phylum (Fig. 2 and Fig. S1). Following the naming convention of MAGs (43), we propose the name “*Candidatus* Marcellius,” after the Marcellus shale formation it was recovered from. The nearest cultivated representative is an obligate aerobe isolated from seawater surrounding hard coral, Coraliomargarita akajimensis strain DSM 45221, sharing 87% 16S rRNA gene sequence identity. Supporting the new genus designation (15), “*Ca.* Marcellius” had a maximum of 52% average amino acid identity (AAI) to all other publicly available *Verrucomicrobia* genomes estimated to be >75% complete (May 2019) (Table S2). Despite a bootstrap value of 44 between “*Ca.* Marcellius” and Coraliomargarita akajimensis in our phylogenetic tree constructed from 11 ribosomal proteins (Fig. 2), these organisms share 48% genome-wide similarity by AAI, well below the genus boundary of 55 to 60% defined in Rodriguez-R et al. (44). Furthermore, analysis using the Genome Taxonomy Database-Tool Kit (GTDB-tk [http://gtdb.ecogenomic.org/]), which assigns taxonomy to MAGs based on a phylogenetic tree of 102 concatenated single-copy marker genes, confirmed our phylogenetic inferences that “*Ca.* Marcellius” is the first representative of a novel genus in the order *Optiutales*.

10.1128/mSphere.00613-19.1 FIG S1 16S rRNA gene phylogenetic tree of the *Verrucomicrobia* phylum, showing the placement of “*Candidatus* Marcellius” in a new genus within the *Opitutales* order. Taxa in boldface represent members of the phylum included in the comparative genomics analysis. Orders (according to SILVA v 132, May 2019) are color-coded and designated by square brackets. *Opitutales* is colored green, *Verrucomicrobiales* red, *Chthoniobacterales* dark blue, *Pedospherales* orange/brown, and *Methylacidiphilales* purple. The order that previously included Kiritimatiella glycovorans strain L21-Fru-AB is now recognized as order *Kiritimatiellae* in the newly designated sister phylum *Kiritimatiekkaeota*, so is not included here. The tree was constructed from an alignment of the 16S rRNA gene using 100 bootstraps. The outgroup is Escherichia coli. The 16S rRNA gene sequences were aligned in Geneious R11 using MUSCLE, concatenated, and a phylogenetic tree was constructed with RAxML 8.2.11. Download FIG S1, JPG file, 2.5 MB. Copyright © 2019 Nixon et al. 2019 Nixon et al. This content is distributed under the terms of the Creative Commons Attribution 4.0 International license.

10.1128/mSphere.00613-19.5 TABLE S2 Details of additional MAGs recovered from hypersaline (H) and deep biosphere (DB) habitats that have been made public since comparative genomics analysis was completed. Listed are the names used in this study, the publications they were reported in (Vavourakis et al. [28], Wu et al. 2017 [26], and Kimbrel et al. [29]), NCBI BioSample accession numbers for MAG sequences, habitat type, estimated completeness, and amino acid average identity (AAI) against the “*Ca.* Marcellius” MAG described in the main text. AAI was calculated as described in Materials and Methods of the main text. *^a^*BioSample numbers in italics have been created for this study to make corresponding MAGs publicly available, with permission from the authors of reference 2. *^b^*Estimated completeness values are as originally published. *, No NCBI BioSample accession number; MAG available at http://downloads.jbei.org/data/microbial_communities/microbial_communities.html. Download Table S2, DOCX file, 0.2 MB. Copyright © 2019 Nixon et al. 2019 Nixon et al. This content is distributed under the terms of the Creative Commons Attribution 4.0 International license.

Like the rest of the *Verrucomicrobia* phylum, members of the *Puniceicoccaceae* family are diverse in range of habitats and metabolisms. Other cultivated strains from this family inhabit seawater (14, 15), marine sand (45), the digestive tract of a marine clamworm (14), and a fermenter (46). The metabolic lifestyles of these cultivated strains range from obligately aerobic (45) to facultatively anaerobic (14), and some are known to perform dissimilatory nitrate reduction (46). To date, six *Verrucomicrobia* MAGs have been recovered from the terrestrial subsurface, three from shallow (5-m) aquifer waters (32) and three from deep (300- to 450-m) granitic groundwaters (26). However, phylogenetic analysis of these MAGs places half of these in the newly recognized sister phylum *Kiritimatiellaeota* (27) and not in the *Verrucomicrobia* phylum itself (Fig. S2). The recovery and analysis of “*Ca.* Marcellius” from the shale gas well reported here therefore extends the distribution of the *Verrucomicrobia* phylum to kilometers below the surface of the Earth and represents the first detailed analysis of a deep-biosphere *Verrucomicrobia* genome.

10.1128/mSphere.00613-19.2 FIG S2 Phylogenetic tree showing the placement of additional hypersaline and deep biosphere MAGs in the *Verrucomicrobia* and *Kirimitiellaeota* phyla, alongside genomes used in comparative genomics analysis. “*Ca.* Marcellius” is highlighted in boldface. Orders (according to Silva v 132, May 2019) are color-coded and designated by square brackets. *Opitutales* is colored green, *Verrucomicrobiales* red, *Chthoniobacterales* dark blue, *Pedospherales* orange/brown, and *Methylacidiphilales* purple. The newly recognized *Kiritimatiellaeota* phylum (27) is shown in black. The outgroup is Escherichia coli. The tree was constructed from a concatenated alignment of 11 conserved ribosomal genes (large subunits L5, L6, L14, L15, L16, L18, L22, and L24 and small subunits S3, S8, and S17) using 100 bootstraps. Gene sequences were aligned in Geneious R11 using MUSCLE, concatenated, and a phylogenetic tree was constructed with RAxML 8.2.11. Download FIG S2, PDF file, 0.2 MB. Copyright © 2019 Nixon et al. 2019 Nixon et al. This content is distributed under the terms of the Creative Commons Attribution 4.0 International license.

### “*Ca.* Marcellius” is the only *Verrucomicrobia* detected in late-stage shale produced fluids.

Given the recovery of the “*Ca.* Marcellius” genome from this shale gas well, we investigated the distribution of *Verrucomicrobia* 16S rRNA genes from our previously published hydraulically fractured shale wells (Table S3). Besides the Marcellus-5 well that “*Ca.* Marcellius” was assembled from, our 16S rRNA gene sampling included 37 time series samples collected from 4 shale wells in the Marcellus and Utica formations (33, 47). Surprisingly, our analysis yielded no evidence of members of the “*Ca.* Marcellius” genus in produced fluids from these other 4 wells. However, we did recover a total of fifteen other *Verrucomicrobia* amplicon sequence variants (ASVs) from two Utica shale gas wells (Utica-2 and Utica-3) (33, 40). These *Verrucomicrobia* were rare members of the community, ranging from 0.1% to 3.6% (average, 1.2%). Notably, these *Verrucomicrobia* were recovered from input and early produced fluids, specifically from flowback fluid collected before 17 days, when salinities were less than 40 g/liter (chloride [35]).

10.1128/mSphere.00613-19.6 TABLE S3 Relative abundances of *Verrucomicrobia* amplicon sequence variants (ASVs) in 16S rRNA datasets from other shale gas wells. Download Table S3, DOCX file, 0.6 MB. Copyright © 2019 Nixon et al. 2019 Nixon et al. This content is distributed under the terms of the Creative Commons Attribution 4.0 International license.

Like “*Ca.* Marcellius,” the majority of the fifteen shale *Verrucomicrobia* ASVs belong to the order *Opitutales.* These were divided equally between the families *Opitutaceae* (5 ASVs) and *Puniceicoccaceae* (5 ASVs). All *Puniceicoccaceae* ASVs had greatest amplicon alignment similarity to marine strain Cerasicoccus frondis YM31-066 (87 to 91% identity over 279 to 396 bp [48]) and a maximum of 84% identity to the 16S rRNA gene sequence recovered in “*Ca.* Marcellius.” Our results indicate that *Verrucomicrobia*, especially members of the *Opitutales*, are often present in low abundance in input fluids but rarely persist in fractured shales beyond the early stages of flowback, when salinities become elevated due to water-rock interactions. “*Ca.* Marcellius” therefore appears to be unique among the *Verrucomicrobia* in its tolerance of the extreme conditions of a fractured shale and can persist hundreds of days postfracturing.

### Members of the *Verrucomicrobia* phylum may play roles in nitrogen cycling.

Analysis of the “*Ca.* Marcellius” genome uncovered the capacity for aerobic respiration (complete TCA cycle, NADH dehydrogenase complex, and an electron transport chain with cytochrome *c* oxidase), yet we failed to detect the functional genes for dissimilatory nitrate (*narGHI* and *napAB*), nitrite (*nirBD* and *nrfAH*), sulfate (*sat*, *aprA*, and *dsrA*), thiosulfate (*psrA*), or tetrathionate (*ttrA*) reduction. Given the absence of detectable oxidized electron acceptors at the time of sampling, the prevalence of ferrous iron in the well (33), and the enrichment and cooccurrence with obligate anaerobes (e.g., fermenters like *Halanaerobium* [34, 47, 49] and methanogens [41]) (Fig. 1b), we infer this organism is likely a facultative anaerobe, with the capacity for anaerobic metabolism via fermentation. While we recognize nonobligate fermentation can be difficult to infer from genome alone, based on the surrounding metabolisms and geochemistry, absence of any anaerobic respiratory metabolisms encoded in the genome, and the capacity for fermentation by other *Verrucomicrobia* (27), we posit that fermentation enables “*Ca.* Marcellius” persistence in the shale ecosystem.

We also recovered evidence that “*Ca.* Marcellius” has genes to fix nitrogen to ammonia via the *nifHDKENB* complement, although we appreciate the presence of genes does not always confer nitrogen fixation ability (50). A number of ammonium-containing additives were included in the injected fluids of the Marcellus-5 shale gas well studied here (33 and www.fracfocus.org). Given the high cost of nitrogen fixation and the abundant fixed nitrogen in the system, we consider it unlikely the organism would be fixing nitrogen at the time sampled. However, if “*Ca.* Marcellius” is able to fix nitrogen, this and other fractured shale taxa may provide an important ecosystem service as these substrates become depleted over time.

We looked for putative nitrogen fixation capacity via the presence of *nif* genes in other nearly complete *Verrucomicrobia* genomes. Completion of these genomes was assessed on the presence of the same core set of single-copy genes used to assess “*Ca.* Marcellius” completion. Aside from the known nitrogen-fixing capacity of acidophilic methanotrophs *Methylacidiphilum infernorum* V4 and *M. fumariolicum* SolV (19, 51), “*Ca.* Marcellius” is the only other *Verrucomicrobia* genome (>75% complete) of the 32 analyzed with the complete *nifHDKENB* complement for nitrogen fixation (Fig. 2). Two less complete genomes not included in the phylum-wide comparison were also found to have all 6 *nif* genes: TH2747, isolated from the nutrient-poor Trout Bog freshwater lake in Wisconsin, USA (2), and *Opitutaceae* bacterium TSB47, recovered from the gastrointestinal tract of a termite. In summary, genomic potential for nitrogen fixation is patchy but encoded across clades within the *Verrucomicrobia* phylum. This potential for nitrogen fixation expands the metabolic repertoire of the *Verrucomicrobia* and indicates these taxa play roles in nitrogen cycling across habitats.

### “*Ca.* Marcellius” has the genomic capacity to gain energy from chemicals injected during hydraulic fracturing.

To analyze possible carbohydrate utilization by “*Ca.* Marcellius,” we inventoried substrates targeted by glycoside hydrolases (GHs), enzymes that hydrolyze glycosidic bonds between two or more carbohydrates. “*Ca.* Marcellius” has a total of 60 GHs inferred to degrade hemicellulose (43 GHs), starch (12), cellulose (4), and chitin (1). Many hemicellulose-specific GHs were annotated as galactosidases (18), fourteen of which belong to the GH family 2. We also detected three acetyl xylan esterase and three endoxylanase genes (GH families 8 and 210), as well as four genes encoding alpha-rhamnosidase (3 from GH family 78). Our results indicate “*Ca.* Marcellius” is able to use a wide range of polysaccharides as substrates but is especially optimized to degrade hemicellulosic polysaccharides.

We examined metabolic overlap between genes in the “*Ca.* Marcellius” genome and common polymers injected during well drilling and hydraulic fracturing processes (52). Cellulose- and starch-based additives are frequent constituents of drilling muds, used to add viscosity to the fluid (53, 54). While we were unable to confirm the composition of the drilling mud used in Marcellus-5, these muds likely contained similar organic polymers (53). Given that large volumes of drilling mud often remain in formation after drilling (54), it is possible that residual starch and cellulose from drilling muds support the growth of microorganisms with genetic machinery like “*Ca.* Marcellius” (53).

Polyacrylamide addition to shale formations is increasing, as this synthetic amide-based polymer is used as a friction reducer in slickwater hydraulic fracturing operations (52). This compound represents the only organic polymer we could confirm was added to the Marcellus-5 well and is known to support microbial growth (55). As such, we looked for evidence that “*Ca.* Marcellius” can degrade polyacrylamide using amidase enzymes that act on the carbon-nitrogen bonds in amide-based polymers. We found evidence for three amidase-encoding genes, suggesting “*Ca.* Marcellius,” like other taxa from fractured shales (40), degrade polyacrylamide.

Guar gum is one of the most volumetrically significant organic polymers added to input fluids and is used in traditional gel-based hydraulic fracturing operations (52). Although not used in hydraulic fracturing of the Marcellus-5 well studied here, we also found evidence that “*Ca.* Marcellius” can fully degrade guar gum (alpha-galactosidase and beta-mannosidase genes) and metabolize mannose, the major sugar that makes up the polymer (glucokinase and phosphomannose isomerase genes). In addition to polyacrylamide degradation, “*Ca.* Marcellius” has the capacity to degrade guar gum and ferment mannose to support growth, similar to other fractured shale taxa (56). Taken together, our findings highlight that degradation of exogenous organic polymers introduced in subsurface engineering operations likely facilitates microbial metabolism and growth in the deep subsurface.

### Hemicellulose degradation is a common trait encoded across the *Verrucomicrobia*.

**FIG 3:**
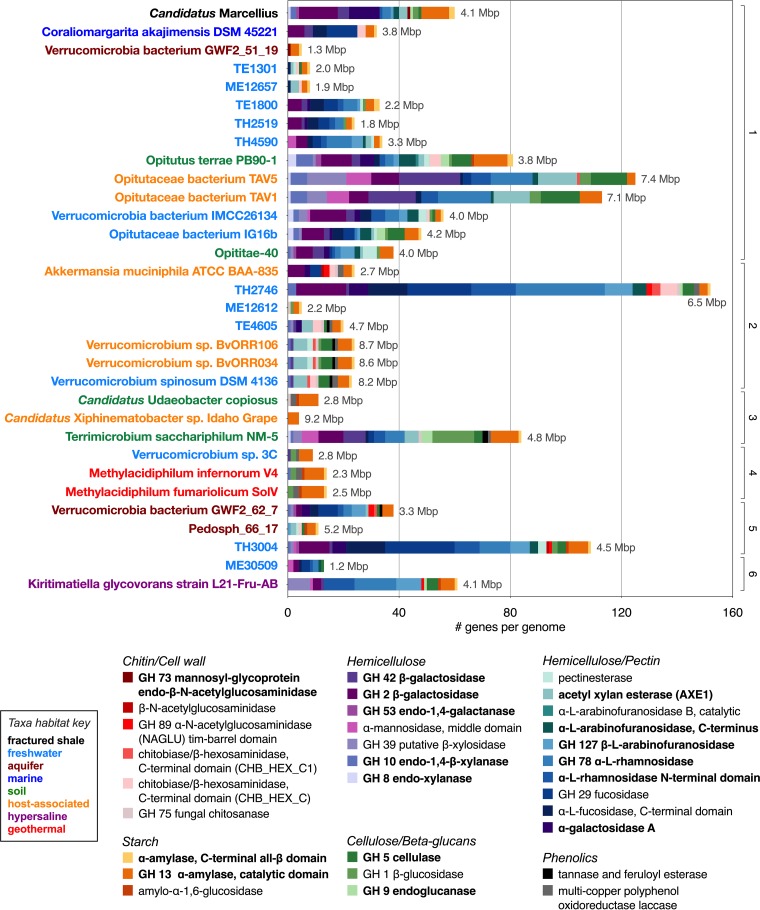
Glycoside hydrolase (GH) genes in “*Candidatus* Marcellius” (boldface) and 31 other nearly complete *Verrucomicrobia* genomes. The “*Ca.* Marcellius” genome is particularly enriched in hemicellulose-degrading genes, a characteristic of other *Opitutales* representatives. Genome size (in megabase pairs, or Mbp) of each *Verrucomicrobia* genome is given in gray text next to each bar. Genomes are grouped by order (1, *Optitulates*; 2, *Verrucomicrobiales*; 3, *Chthoniobacterales*; 4, *Methylacidiphilales*; 5, *Pedospherales*; and 6, the formerly acknowledged subdivision 5, the only cultivated member of which [Kiritimatiella glycovorans strain L21-Fru-AB] was recently assigned to the novel sister phylum *Kiritimatiellaeota* [27]). Taxon names are color coded for their habitat of origin.

Given the potential importance of polysaccharide degradation to the establishment of “*Ca.* Marcellius” in the fractured shale environment, we assessed this trait across *Verrucomicrobia* genomes from a diverse range of habitats (Fig. 3). Like many bacteria, all *Verrucomicrobia* genomes included in our analyses have the capacity for starch degradation. Notably absent from *Verrucomicrobia* genomes sampled were annotated genes for polyphenolic degradation, suggesting this metabolism is not a widely encoded function. GH enzymes common to the *Verrucomicrobia* include GH 5, GH 2, and GH 1, all common GH families with multiple functional capacities. Supporting our findings from a single genome, genes for hemicellulose degradation were by far the most enriched in genomes across the phylum. We recovered an average of 27 hemicellulytic GH genes/genome, with genes for cleavage of galactose monomers (GH 42) and xylose monomers (GH 10) being the most prevalent. Given that hemicellulose, a critical component of plant biomass, is one of the most abundant polymers on Earth (57), degradation of this compound could explain *Verrucomicrobia* persistence across host, soil, aquatic, and subsurface environments.

Habitat of origin does not appear to be linked to carbohydrate-degrading capacity in the *Verrucomicrobia*, as genomes with the most and least GHs were found in similar habitat types (Fig. 3 and Fig. S3). Generally, the smaller genomes are less enriched in GHs and the larger genomes more enriched. However, the largest genomes among those sampled here carried some of the smallest GH gene sets, notably “*Candidatus* Xiphinematobacter sp. strain Idaho Grape,” with only 4 GHs found in a genome 9.2 Mbp in length (Fig. 3 and Fig. S3). In contrast, we did find patterns of glycoside hydrolase number associated with taxonomy, regardless of genome size. In particular, *Opitutales* has the most genomes enriched in hemicellulose-degrading genes, including the novel genome “*Ca.* Marcellius” described here (Fig. 3). Furthermore, clusters of closely related genomes in subdivisions 1 and 7 share strikingly similar patterns of polysaccharide-degrading genes, regardless of their habitat of origin. For instance, host-associated genomes of *Verrucomicrobium* sp. strains BvORR106 and BvORR034 share glycoside hydrolases almost identical to that of the genome of freshwater-derived Verrucomicrobium spinosum DSM 4136 (Fig. 3). Similarly, genomes of acidophilic methanotrophs Methylacidiphilum infernorum V4 and *M. fumariolicum* SolV carry a set of starch- and cellulose-degrading genes similar to that of freshwater-derived *Verrucomicrobium* sp. strain 3C (Fig. 3). These findings suggest that the ability to degrade polysaccharides is more conserved along phylogenetic, rather than habitat, boundaries.

10.1128/mSphere.00613-19.3 FIG S3 Number of glycoside hydrolase genes (GHs) in each *Verrucomicrobia* genome included in comparative analysis, plotted against genome size (in mega base pairs, Mbp). The genomes with the highest and lowest numbers of GHs are named. Genomes are color-coded according to their habitat of origin, as described for Fig. 3. Download FIG S3, PDF file, 0.04 MB. Copyright © 2019 Nixon et al. 2019 Nixon et al. This content is distributed under the terms of the Creative Commons Attribution 4.0 International license.

Despite the relatively limited genome sampling of this phylum, the capacity to degrade polysaccharides is emerging as a widely acknowledged trait among *Verrucomicrobia* genomes (2, 4, 5, 58). In agreement with our phylum-wide assessment (Fig. 3), various degrees of GH enrichment have been reported for less complete genomic representatives not included in our analysis. For example, Martinez-Garcia et al. used fluorescently labeled substrates, fluorescence-activated cell sorting, and single-cell genomics to identify 5 *Verrucomicrobia* genotypes that were active in polysaccharide degradation (59). These *Verrucomicrobia* genomes were found to be enriched in a wide spectrum of GHs and likely made a disproportionate contribution to active xylan degradation despite their low relative abundance in the community (59). In a similar fashion, it is possible that the low-abundance “*Ca.* Marcellius” organism plays a critical role in carbon processing in the fractured shale ecosystem.

These prior studies, when considered alongside our own assessment of polysaccharide degradation potential, provide compelling evidence that *Verrucomicrobia* are widespread contributors to the cycling of carbon. Microorganisms that degrade heterogeneous, polymeric organic matter, such as biological polysaccharides, act as gatekeepers to the microbial carbon cycle, providing downstream monomers (e.g., hexose or pentose) that serve as substrates for lower trophic levels (57). To date, our knowledge of *Verrucomicrobia* ecology is largely limited to freshwater and soil ecosystems. While these environments represent significant contributors to the global carbon cycle, our recovery of “*Ca.* Marcellius” from a deep rock-hosted, hypersaline ecosystem demonstrates that members of *Verrucomicrobia* extend across a greater range of habitats than previously considered. As such, the magnitude and contribution of *Verrucomicrobia* to the carbon cycle is only beginning to be appreciated.

### Tolerance to environmental conditions may enable *Verrucomicrobia* to persist in the deep subsurface.

**FIG 4:**
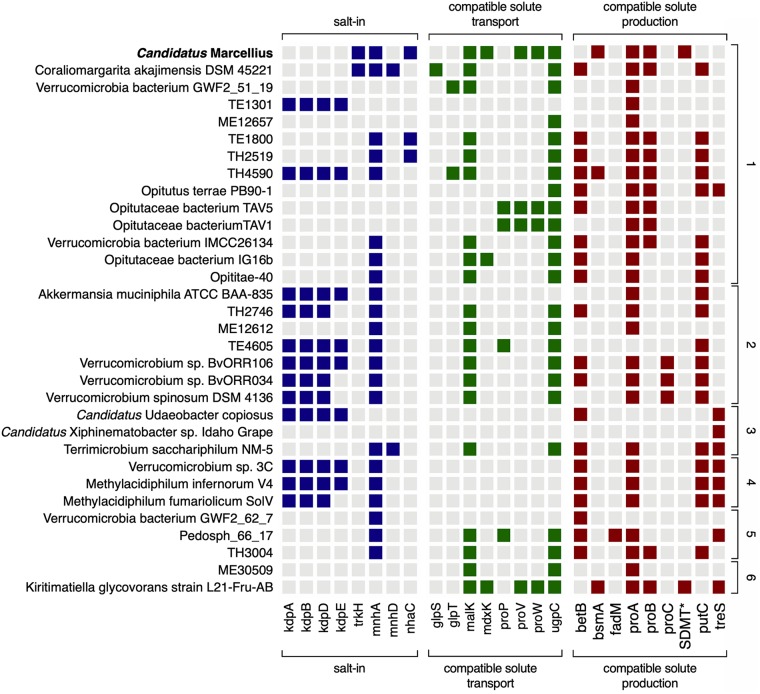
Genes encoding the compatible solute osmoprotectant strategy are common in *Verrucomicrobia*. Osmoprotectant genes detected in “*Ca.* Marcellius” (boldface) are compared to the 31 other nearly complete *Verrucomicrobia* genomes, grouped by order (1, *Optitulates*; 2, *Verrucomicrobiales*; 3, *Chthoniobacterales*; 4, *Methylacidiphilales*; 5, *Pedospherales*; and 6, formerly acknowledged subdivision 5, the only cultivated member of which [Kiritimatiella glycovorans strain L21-Fru-AB] was recently assigned to the novel sister phylum *Kiritimatiellaeota* [27]). Genes are listed on the *x* axis, and genomes are listed on the *y* axis. Colored boxes indicate the presence of that gene in the genome. The different strategies for osmoprotection are listed at the top and are represented by color (salt-in, blue; osmolyte transport, green; osmolyte production, red). *, SDMT, sarcosine/dimethylglycine *N*-methyltransferase. All other gene descriptions are given in Table S4.

The “*Ca.* Marcellius” genome was only detected between 250 and 313 days after fracturing, when TDS measured 104 g/liter on average over the period sampled, suggesting this organism should have mechanisms for salinity tolerance. Genome analysis for osmoprotection strategies (Table S4) revealed the presence of genes for both salt-in and osmolyte salt tolerance mechanisms (60). First, “*Ca.* Marcellius” contained genes for the salt-in strategy (*nhaC*, *mnhA*, *phaD*, and *trkH*), whereby ions like potassium accumulate in the cell to balance with the surrounding environment (Fig. 4). “*Ca.* Marcellius” also has genomic potential to employ the compatible solute method, whereby an organic molecule (e.g., amino acid or sugar derivative) accumulates by transport or *de novo* synthesis to maintain osmotic pressure within the cell. “*Ca.* Marcellius” has the capacity to take up but not degrade known osmolytes, including proline (*proW* and *proV*), suggesting this compound can be transported intracellularly for osmoprotection. We also note that it is possible that proline could be incorporated into proteins, as its sole use as an osmoprotectant is not possible to determine from genome sequence alone. The genome also carries genes encoding transporters for osmoprotectants like maltose (*malK*) and glycerol-3-phosphate (*upgC*); however, given that genes for the metabolism of these compounds are also present, use of these compounds in osmoprotection is challenging to infer. This genome also has the capacity to synthesize nitrogenous osmolytes like (i) proline from glutamate (*proAB*) and (ii) glycine betaine from glycine (*bsmA*, *sarcosine/dimethylglycine N-methyltransferase*).

10.1128/mSphere.00613-19.7 TABLE S4 Osmoadaptation genes screened for in comparative genomics analysis. Only genes with positive hits are shown in Fig. 4. Download Table S4, DOCX file, 0.4 MB. Copyright © 2019 Nixon et al. 2019 Nixon et al. This content is distributed under the terms of the Creative Commons Attribution 4.0 International license.

Nuclear magnetic resonance analyses of metabolites from forty different shale-produced fluids support the osmoprotectant strategies identified in the “*Ca.* Marcellius” genome. For instance, metabolite data have demonstrated that glycine and glycine betaine were widely recovered in shale-derived produced fluids, including those from the Marcellus-5 well at the time “*Ca.* Marcellius” was recovered (33). Moreover, the capacity for shale microorganisms, like *Methanohalophilus*, to synthesize glycine betaine has been shown in laboratory microcosms (33, 41). When released into the surrounding environment (e.g., by viral lysis), these compounds can be used as growth substrates, sustaining a metabolic network composed of key shale members, including *Halanaerobium* (33). Based on analyses presented here, we further entertain the possibility that “*Ca.* Marcellius” could contribute to *in situ* synthesis of critical osmolytes/substrates.

Other *Verrucomicrobia* genomes have been recovered from hypersaline environments. The first hypersaline *Verrucomicrobia* genome was an isolate, Kiritimatiella glycovorans strain L21-Fru-AB (21), recovered from a photosynthetic mat (salinity of 170 g/liter at the time of sampling) (61). While it was originally posited, in part due to the lack of other saline-tolerant *Verrucomicrobia*, that strain L21-Fru-AB should be a new phylum (*Kiritimatiellaeota*) (27), we have included it in our phylum-wide analysis given its similarly hypersaline habitat of origin. In line with their recovery from environments with similar salinities, “*Ca.* Marcellius” (116 g/liter TDS) and *K. glycovorans* genomes share nearly the same osmoprotectant gene profile (10/11 genes), a gene pattern not conserved across the remaining *Verrucomicrobia* genomes (Fig. 4). Interestingly, both saline-derived genomes contain a glycine betaine synthesis pathway, suggesting this pathway is an important trait for *Verrucomicrobia* residing in hypersaline habitats.

Our comparative genome analyses revealed osmoadaptation is broadly encoded across other *Verrucomicrobia* subdivisions. We found no correlation between osmoprotectant gene number and habitat origin or phylogeny. For instance, the number of osmoprotectant genes in freshwater environments range from two (ME12756) to thirteen (TH4590) genes and those associated with hosts range from one (“*Candidatus* Xiphinematobacter sp. strain Idaho Grape”) to eleven (*Verrucomicrobium* sp. strain BvORR106). Osmoprotectant strategies like those we report here enable more than salinity adaptation, they also allow microorganisms to withstand osmotic stressors like those caused by desiccation in wet and dry seasons in soils (62) and along brackish coastal estuaries. Therefore, resistance to environmental perturbations may allow members of this phylum to colonize and withstand dynamic habitats like those in fractured shales, the gut, and soils.

A large number (32) of *Verrucomicrobia* MAGs were recently recovered from soda lake sediments (28) and a hypersaline pond (29). The salinity of these environments ranges from 42.9 to 110 g/liter TDS, similar to that from which the production fluids of “*Ca.* Marcellius” were recovered. These MAGs were not publicly available at the time we conducted the phylum-wide comparative genomics analyses reported above. We calculated similarity to “*Ca.* Marcellius” using AAI (Table S2) and constructed an additional phylogenetic tree (Fig. S2). The closest AAI identity is 52% (CSSed10_295, 88% complete [28]), placing it within the *Puniceicocacceae* family but not within the “*Candidatus* Marcellius” genus. Nine other hypersaline MAGs clade with “*Ca.* Marcellius” in the *Opitutales* order (Fig. S2); however, the majority appear to belong to the newly established sister phylum *Kiritimatiellaeota* and, hence, are no longer recognized as *Verrucomicrobia* according to SILVA taxonomy (release 132) (3). Nevertheless, the recovery of a large number of MAGs belonging to *Verrucomicrobia* and its sister phylum from hypersaline environments suggests a more widespread tolerance to high salinity than previously appreciated. The recovery of “*Ca.* Marcellius” from hydraulic fracturing production fluids therefore extends not only the environmental distribution but also the salinity tolerance of the *Verrucomicrobia* phylum.

### Evidence for *Verrucomicrobia*-virus interactions.

Our prior research (33, 34, 47) suggests viral immunity is an important factor to persisting in shale ecosystems. Evidence for this includes (i) the recovery of CRISPR-associated proteins in all persisting genomes (34), (ii) active spacer incorporation within CRISPR-Cas arrays from genomes sampled over time in produced fluids (34, 47), and (iii) expressed CRISPR-Cas genes in response to active viral predation (33). Here, we explore the possible relationships between viruses and members of the *Verrucomicrobia*.

We mined the nearly complete *Verrucomicrobia* genomes for the presence of CRISPR-Cas systems and found that CRISPR-Cas immunity was present in 32% of the genomes. Of the CRISPR-Cas systems that could be classified, we recovered both types I and III. Interestingly, only *Verrucomicrobium* sp. strain BvORR034 (from the root of a sugar beet) and “*Ca.* Marcellius” contained both types. Within “*Ca.* Marcellius” we detected six CRISPR-Cas arrays containing a total of 189 spacers. We previously created a database of viruses from hydraulically fractured shale wells consisting of 1,838 viral genomes from 40 metagenomes (47) and found that none of the 189 “*Ca.* Marcellius” spacers matched this viral database. This indicates that “*Ca.* Marcellius” experienced viral predation in a different habitat before its recovery from the hydraulically fractured well or that the viruses preying on “*Ca.* Marcellius” were in too low abundance to be sampled by our efforts. Further, no spacer matches from the “*Ca.* Marcellius” genome to viral sequences in the NCBI RefSeq database were found. We must consider, however, that like the host genomes of this phylum, *Verrucomicrobia* viruses are likely also undersampled. Taken together, it appears that “*Ca.* Marcellius” has the genomic mechanisms to withstand the viral predation known to be active in fractured shale ecosystems.

Despite the presence of a functional CRISPR-Cas-acquired immune system in the “*Ca.* Marcellius” genome, we also detected genes indicative of prophage or viruses integrated into the host genome (Table 1). A genome-based network analysis of shared protein content classified these as members of the double-stranded DNA viral order *Caudovirales*. These two prophage regions were located on scaffold_15 and scaffold_45, spanning ∼39 kbp and ∼18 kbp, respectively, when analyzed using VirSorter (63, 64). Examination of these genomic regions indicate that they contain multiple genetic defects and are remnants of degraded prophage, lacking genes required for phage integration, induction, and propagation (65). For example, the defective prophage region on scaffold_15 contains only three genes annotated as phage: two Mu-like phage proteins and a large terminase subunit (genes 32 to 34). Flanking the phage proteins are a series of short genes annotated as unknown function or hypothetical proteins (∼300 to 450 bp), with best gene hits to *Verrucomicrobia* genomes. The defective prophage region on scaffold_45 contains a single gene annotated as phage, a 281-bp fragment of a terminase large subunit, and six sequential transposase genes (genes 2 to 7). Collectively, we instead posit that these regions are likely rapidly evolving parts of the genome due to interactions with mobile genetic elements, including prophage.

**TABLE 1:** Summary of prophage and CRISPR-Cas systems recovered from *Verrucomicrobia* genomes

Genome	Order^*a*^	No. of prophage	CRISPR type^*b*^	No. of loci	No. of spacers
“*Candidatus* Marcellius”	1	2	I and III	6	189
Coraliomargarita akajimensis DSM 45221	1				
*Verrucomicrobia bacterium* GWF2_51_19	1				
TE1301	1				
ME12657	1				
TE1800	1				
TH2519	1				
TH4590	1				
Opitutus terrae PB90-1	1	3			
*Opitutaceae bacterium* TAV5	1	2	I	2	137
*Opitutaceae bacterium* TAV1	1	7	I	2	130
*Verrucomicrobia bacterium* IMCC26134	1	3	ND	1	29
*Opitutaceae bacterium* IG16b	1				
Opititae-40	1				
Akkermansia muciniphila ATCC BAA-835	2	1	ND	2	11
TH2746	2				
ME12612	2				
TE4605	2				
*Verrucomicrobium* sp. strain BvORR106	2	3	ND	1	61
*Verrucomicrobium* sp. BvORR034	2	4	I and III	1	12
Verrucomicrobium spinosum DSM 4136	2	4	III	2	84
“*Candidatus* Udaeobacter copiosus”	3				
“*Candidatus* Xiphinematobacter sp. strain Idaho Grape”	3				
Terrimicrobium sacchariphilum NM-5	3	2			
*Verrucomicrobium* sp. 3C	4		I	1	35
*Methylacidiphilum infernorum* V4	4		ND	2	11
*Methylacidiphilum fumariolicum* SoIV	4		III	2	33
*Verrucomicrobia bacterium* GWF2_62_7	5				
Pedosph_66_17	5				
TH3004	5	1			
ME30509	6				
Kiritimatiella glycovorans strain L21-Fru-AB	6		ND	3	67

a 1, *Optitulates*; 2, *Verrucomicrobiales*; 3, *Chthoniobacterales*; 4, *Methylacidiphilales*; 5, *Pedospherales*; 6, formerly acknowledged subdivision 5, the only cultivated member of which (Kiritimatiella glycovorans strain L21-Fru-AB) was recently assigned to the novel sister phylum *Kiritimatiellaeota* (27).

b ND, the CRISPR-Cas type was unable to be determined from annotated genes.

### Conclusions.

We recovered the genome of a novel genus of *Verrucomicrobia*, “*Candidatus* Marcellius”, from a produced water sample collected 313 days after hydraulic fracturing of a Marcellus shale gas well. Phylogenetic analyses placed “*Ca.* Marcellius” within a novel genus of the *Opitutales* order of the *Verrucomicrobia* phylum. “*Ca.* Marcellius” is the first member of the phylum to be recovered from a kilometers-deep environment in the deep terrestrial subsurface. Additionally, this genome is one of a small number of *Verrucomicrobia* genomes recovered from a hypersaline habitat. Our genomic assessment suggests members of the *Verrucomicrobia* occupy a wider array of environmental niches than previously appreciated.

Analysis of “*Ca.* Marcellius” indicated it has the capacity to degrade polymeric carbon substrates, including the widely used hydraulic fracturing fluid additives guar gum and polyacrylamide. We also found evidence for nitrogen fixation, osmoadaptation, and viral immunity, all traits we propose enable this organism to colonize a deep, rock-hosted habitat. Our phylum-wide comparative genomic analyses revealed that hemicellulosic degradation and osmoprotectant production were encoded broadly across *Verrucomicrobia* from freshwater, marine, soil, host-associated, aquifer, and geothermal environments. In spite of their broad environmental distribution, much of *Verrucomicrobia* phylogenetic and physiological diversity remains undersampled today, signifying that *Verrucomicrobia* contributions to carbon and nitrogen cycling are much greater than our current understanding suggests. Investigations of genomic attributes of organisms capable of withstanding extreme environments like those reported here may ultimately help better constrain the limits to life here on Earth and beyond.

## MATERIALS AND METHODS

### Production fluid collection and geochemistry.

Shale-produced fluids were collected from gas-fluid separators of a hydraulically fractured shale well (Marcellus-5) from the Marcellus Shale Energy and Environment Laboratory (MSEEL) in the Marcellus shale play. The well was shut in for a period of 33 days after hydraulic fracturing, with production commencing from day 34. Sample days used in this study refer to the number of days since hydraulic fracturing, inclusive of this shut-in period. Fluid-dissolved anions (SO_4_^2−^, F^−^, Cl^−^, Br^−^, NO_3_^−^) were analyzed using a ThermoScientific Dionex ICS-2100 ion chromatograph. Samples for ion chromatography were diluted 10- to 100-fold due to the high salinity. Major and trace cations (Na, Mg, K, Ca, Si, Fe, Mn, Ba, and Li) were acidified immediately after filtration to ∼0.5% with nitric acid and subsequently analyzed using a Perkin-Elmer Optima 4300DV inductively coupled plasma optical emission spectrometer. Ammonia (NH_3_ + NH_4_^+^) was analyzed using a Skalar San++ nutrient analyzer using a modified Bertholet method. Geochemistry of the fluid was analyzed and used to calculate total dissolved solids (TDS).

### Metagenomic sequencing and assembly.

For genomic sample collection, 300 to 1,000 ml of production fluids (collected 103, 118, 152, 250, and 313 days after hydraulic fracturing) were concentrated onto 0.22-μm-pore-size polyethersulfone (PES) filters (Millipore, Fisher Scientific). Total nucleic acids were extracted from the filter using a modified phenol-chloroform extraction (66). DNA was submitted for sequencing at the Joint Genome Institute. Libraries were prepared and quantified using an Illumina library creation kit (KAPA Biosystems) with solid-phase reversible 402 immobilization size selection. Libraries were sequenced on the Illumina HiSeq 2500 sequencing platform utilizing a TruSeq rapid paired-end 404 cluster kit. Raw sequencing data were trimmed from both the 5′ and 3′ ends using Sickle (https://github.com/najoshi/sickle). Each sample then was assembled individually using IDBA-UD (33, 34, 57, 67) with default parameters.

### Metagenomic annotation and binning.

All scaffolds of ≥5 kb were included when binning genomes from the assembly. For each metagenome, we obtained the genome-resolved bins using a combination of phylogenetic signal, coverage, and GC content (34, 67, 68; http://github.com/TheWrightonLab/metagenome_analyses). This pipeline produces a summary of scaffold statistics (GC content, coverage, and length), in addition to the top hit for gene product and taxon against the UniRef90 protein database for every gene on the scaffolds. Scaffolds were binned based on a combination of GC content, coverage, and taxonomic signals. Details of software dependencies and scripts for the pipeline (quicklooks) used to obtain these metrics can be found on the Wrighton Lab github page (http://github.com/TheWrightonLab/metagenome_analyses). Genome completion was estimated based on the presence of core gene sets (highly conserved genes that occur in single copy) for *Bacteria* (31 genes) and *Archaea* (104 genes) using Amphora2 (69). Overages (gene copies of >1 per bin), indicating potential misbins, along with GC and phylogeny, were used to manually remove potential contaminations from the bins using the same quicklooks output that scaffolds were binned from. More rigorous annotation and phylogenetic analyses were subsequently conducted on resulting bins, as outlined below. NCBI accession numbers for MAGs and isolate genomes used in this study can be found in Table S5 in the supplemental material.

10.1128/mSphere.00613-19.8 TABLE S5 Details of publicly available *Verrucomicrobia* genomes considered for comparative genomics analyses. Only genomes 75% complete or higher (based on single copy gene analysis; see Materials and Methods) and which contained the 11 ribosomal proteins used to construct the concatenated ribosomal protein phylogenetic tree (Fig. 2) were included in comparative analyses. Download Table S5, DOCX file, 0.5 MB. Copyright © 2019 Nixon et al. 2019 Nixon et al. This content is distributed under the terms of the Creative Commons Attribution 4.0 International license.

Scaffolds were annotated as outlined in Daly et al. (34). Briefly, open reading frames were predicted using MetaProdigal (70), and sequences were compared using USEARCH (71) to KEGG, UniRef90, and InterproScan (72), with single and reverse best hit (RBH) matches greater than 60 bits reported. The collection of annotations for a protein were ranked. Reciprocal best BLAST hits with a bit score of >350 were given the highest rank (A), followed by reciprocal best BLAST hit to UniRef with a bit score of >350 (B), BLAST hits to KEGG with a bit score of >60 (C), and UniRef90 with a bit score greater than 60 (C). The next rank represents proteins that only had InterproScan matches (D). The lowest rank comprises hypothetical proteins, with only a prediction from Prodigal and bit score of <60.

Near-full-length ribosomal 16S rRNA gene sequences were reconstructed from unassembled Illumina reads using EMIRGE (73). To reconstruct 16S rRNA genes, we followed the protocol with trimmed paired-end reads where both reads were at least 20 nucleotides used in inputs and 50 iterations. EMIRGE sequences were chimera checked before phylogenetic gene analyses. Taxonomic placement of the genome bins relied on the phylogenetic analyses of 16S rRNA genes and ribosomal proteins. Several high-quality bins representative of each taxa persisting 313 days posthydraulic fracturing were selected for manual curation. Genome statistics were calculated using the Distilled and Refined Annotation of MAGs (DRAM) tool (https://github.com/shafferm/DRAM).

### Phylogenetic and metabolic analyses.

Ribosomal gene sequences for ribosomal proteins L5, L6, L14, L15, L16, L18, L22, and L24 and S3, S8, and S17 were aligned using MUSCLE, concatenated in Geneious R7, and run through ProtPipeliner, an in-house python script developed for generation of phylogenetic trees (https://github.com/lmsolden/protpipeliner), to construct the ribosomal phylogenetic tree (Fig. 2). A 16S rRNA gene phylogenetic tree (Fig. S1) was constructed from genes obtained from at least two representatives of each genus across the phylum from SILVA (release 132) (3). 16S rRNA gene sequences were aligned in Geneious R11 using MUSCLE, and a phylogenetic tree was constructed with RAxML 8.2.11 (general time-reversible [GTR] gamma nucleotide model, 999 bootstrap replicates). To place additional hypersaline and deep biosphere MAGs in the *Verrucomicrobia* and *Kiritimatiellaeota* phyla (Fig. S2), ribosomal gene sequences (as described above) from these MAGs and genomes used in comparative genomics analysis were aligned using MUSCLE and concatenated in Geneious R11, and a phylogenetic tree was constructed with RAxML 8.2.11 (GTR gamma nucleotide model, 999 bootstrap replicates). Average amino acid identity (AAI) was calculated using the AAI matrix tool (http://enve-omics.ce.gatech.edu/g-matrix/).

Read-mapped abundance of binned genomes was calculated at the 5 time points using the *rpsC* gene recovered from each bin. This gene was used to track strain resolved abundance patterns across the hydraulically fractured shale metagenomes, as outlined in Borton et al. (33). Briefly, all annotated *rpsC* genes from the five metagenomes were pulled to build an *rpsC* database. Bowtie2 (74) was used to map metagenomic reads by sample to the *rpsC* databases with zero mismatches. Strain-resolved relative abundance was obtained by quantifying the percentage of total reads that mapped, divided by the length of the sequence, and then normalizing to within each sample. Strains included in this analysis were required to have 95% of the *rpsC* sequence covered with mapped reads. Details of *rpsC* read-mapped abundances of genome bins can be found in Table S1.

Given the hypersaline nature of the produced fluids, we assessed the genomes of “*Ca.* Marcellius” and other members of the *Verrucomicrobia* phylum for evidence of osmoprotectant strategies. The full list of osmoprotectant genes we screened for can be found in Table S4. Osmoprotectant genes were queried against the recovered genomes using BLASTp (75), with only matches achieving a bit score of ≥200 considered homologs.

Metabolic profiling was conducted using KEGG KAAS (76) and by manual analyses. For key functional genes, we used a list and homology-based approach to help annotate genes using a bit score threshold of 200. For polysaccharide degradation potential, identified glycoside hydrolases (GH) were identified using InterProScan against the Pfam database. GHs were sorted into functional classes (for example, chitin, hemicellulose, and starch) as previously described (57). Necessary scripts and analyses to perform metagenomic assembly, EMIRGE, and annotation and single-copy genes described here can be accessed from github (http://github.com/TheWrightonLab/metagenome_analyses). Whole-genome taxonomic assignment of “*Ca.* Marcellius” and *K. glycovorans* strain L21-Fru-AB was performed using the Genome Taxonomy Database-Tool Kit (GTDB-tk, Release 03-RS86, 19 August 2018; http://gtdb.ecogenomic.org/).

### Prophage and CRISPR identification.

Prophage were identified in all assembled metagenomes using VirSorter (63, 64), hosted on the CyVerse discovery environment (77). VirSorter was run with default parameters using the virome database, and prophage sequences with category 4 and 5 status were retained. The CRISPR Recognition Tool plugin (CRT; version 1.2) in Geneious was used to identify CRISPR arrays in *Verrucomicrobia* MAGs and isolate genomes. CRISPR-Cas systems were classified by manually examining the CRISPR-Cas genes of annotated contigs (78). A network-based protein classification was used to taxonomically place the shale-derived viral sequences in the context of known viruses according to the ICTV (79, 80).

### Comparative genomics across the *Verrucomicrobia* phylum.

For cross-phylum comparisons at the genome level, a total of 72 additional *Verrucomicrobia* genomes were acquired through the JGI IMG and NCBI genome databases and annotated and assessed for completion (January 2018). We imposed a ≥75% genome completion threshold on genomes to be included for additional analyses. This threshold reduced the number of additional genomes to 31, which were subject to further metabolic and viral analyses as described above. The full list of genomes included in this analysis and their accession numbers are given in Table S5.

A number of additional *Verrucomicrobia* MAGs, recovered from deep biosphere (26) and hypersaline habitats (28, 29), were published after comparative genomics analysis was completed (January 2018). To ensure these highly relevant MAGs were not overlooked in our study, we calculated AAI on those >75% complete (Table S2) and constructed a concatenated ribosomal protein tree (using ribosomal proteins L5, L6, L14, L22, and L24 and S3, S8, and S17; Fig. S2) to resolve their phylogenetic relationships to “*Ca.* Marcellius.” The three deep-biosphere MAGs (26) were not publicly available independent of the metagenomes they were recovered from. With permission from the authors of that study, we make them publicly available here (see italicized accession numbers in Table S2).

### Availability of data and material.

Details of all data generated or analyzed during this study, and where to access them, are included in this article. Sequencing data generated in this study have been deposited in the NCBI sequence read archive under the BioProject numbers PRJNA363633, PRJNA512237, and PRJNA295907. Scripts for metagenomic assembly, EMIRGE, annotation, and single-copy genes described here can be accessed from github (http://github.com/TheWrightonLab/metagenome_analyses). Details of metagenome-assembled genome bin *rpsC* abundances are given in Table S1. Accession numbers and AAI values for the additional hypersaline and deep-biosphere MAGs compared with “*Ca.* Marcellius” are provided in Table S2. Relative abundances of amplicon sequence variants detected in other previously published 16S rRNA gene data sets from other shale gas wells is given in Table S3. Details of osmoadaptation genes screened for are given in Table S4. Accession numbers and completeness/contamination estimates for publicly available *Verrucomicrobia* genomes considered for comparative genomic analysis can be found in Table S5. A 16S rRNA gene phylogenetic tree of the *Verrucomicrobia* phylum, showing placement of “*Candidatus* Marcellius,” is given in Fig. S1. A ribosomal protein tree of these additional MAGs, along with other members of the *Verrucomicrobia* phylum included in comparative genomics analyses, is given in Fig. S2. A plot to show the number of glycoside hydrolases in each genome against genome size is given in Fig. S3.
