# Supplementary material for: Genome-Resolved Metagenomics Extends the Environmental Distribution of the Verrucomicrobia Phylum to the Deep Terrestrial Subsurface
Source: mSphere. 2019 Dec 18;4(6):e00613-19. doi: 10.1128/mSphere.00613-19 (PMC6920513; doi:10.1128/mSphere.00613-19)
Supplement: TABLE S1 [file mSphere.00613-19-st001.docx]

*represents the sum of S3 abundances that could be attributed to the taxonomic assignment given in the table
