# Supplementary figures and images for: Genome-Resolved Metagenomics Extends the Environmental Distribution of the Verrucomicrobia Phylum to the Deep Terrestrial Subsurface
Source: mSphere. 2019 Dec 18;4(6):e00613-19. doi: 10.1128/mSphere.00613-19 (PMC6920513; doi:10.1128/mSphere.00613-19)

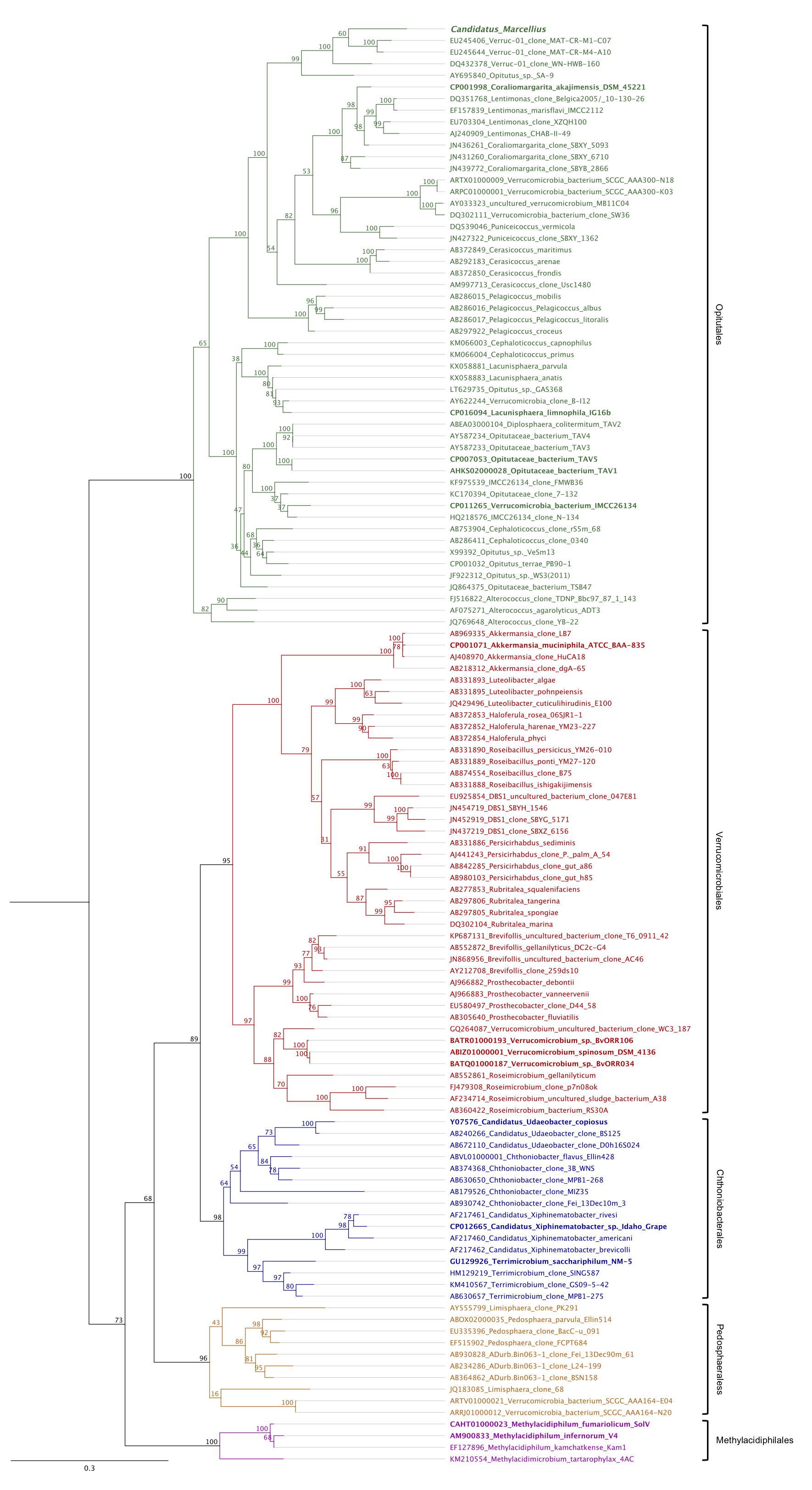

Supplement: FIG S1 [file mSphere.00613-19-sf001.jpg]

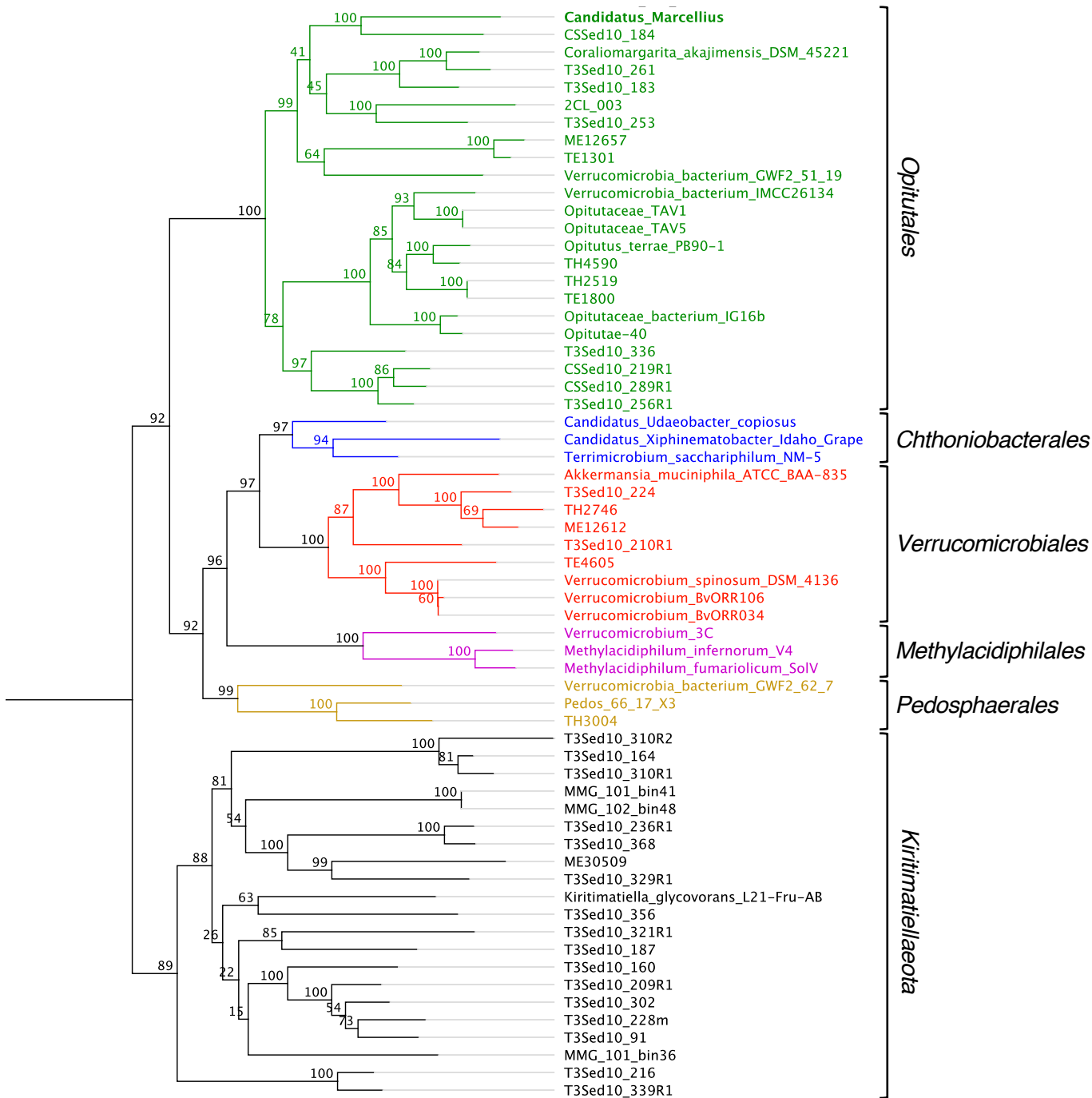

Supplement: FIG S2 [file mSphere.00613-19-sf002.pdf]

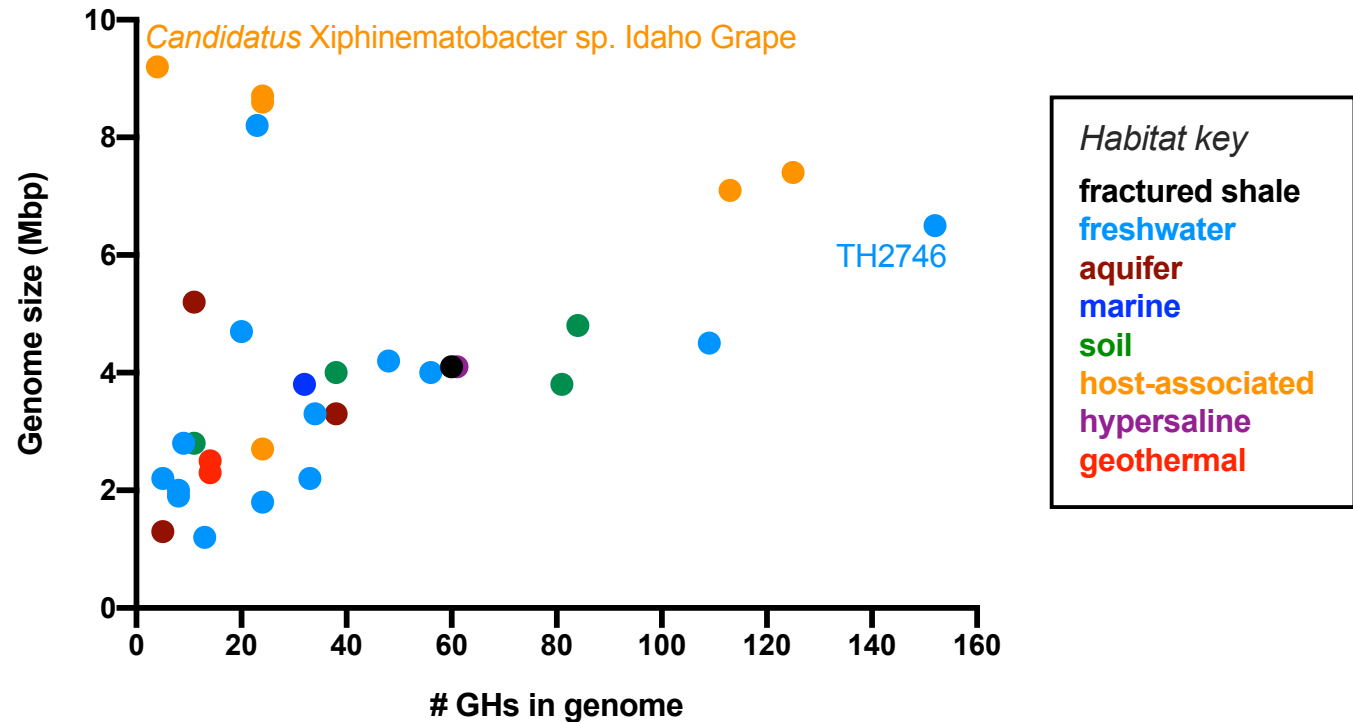

Supplement: FIG S3 [file mSphere.00613-19-sf003.pdf]
