# Supplementary material for: Genome-Resolved Metagenomics Extends the Environmental Distribution of the Verrucomicrobia Phylum to the Deep Terrestrial Subsurface
Source: mSphere. 2019 Dec 18;4(6):e00613-19. doi: 10.1128/mSphere.00613-19 (PMC6920513; doi:10.1128/mSphere.00613-19)
Supplement: Supplementary file 3 [file mSphere.00613-19-st002.docx]

MAG	Ref	NCBI BioSample[a]	Habitat	Completeness[b] (%)	AAI vs *Ca.* Marcellius (%)
CSSed10_289R1	[1]	SAMN08574537	H	99	47
T3Sed10_91	[1]	SAMN08575029	H	96	40
T3Sed10_160	[1]	SAMN08574860	H	95	39
T3Sed10_329R1	[1]	SAMN08574969	H	95	38
CSSed10_219R1	[1]	SAMN08574493	H	94	47
MMR_101_bin41	[2]	*SAMN12606108*	DB	94	37
MMG_102_bin48	[2]	*SAMN12606109*	DB	94	37
T3Sed10_261	[1]	SAMN08574929	H	93	47
T3Sed10_339R1	[1]	SAMN08574976	H	93	40
CSSed10_184	[1]	SAMN08574474	H	91	50
T3Sed10_336	[1]	SAMN08574974	H	90	47
T3Sed10_164	[1]	SAMN08574863	H	89	38
T3Sed10_310R1	[1]	SAMN08574958	H	89	38
T3Sed10_321R1	[1]	SAMN08574965	H	89	39
CSSed10_295	[1]	SAMN08574541	H	88	52
T3Sed10_253	[1]	SAMN08574922	H	88	47
T3Sed10_216	[1]	SAMN08574901	H	87	40
T3Sed10_302	[1]	SAMN08574955	H	87	39
MMR_101_bin36	[2]	*SAMN12606110*	DB	86	38
T3Sed10_236R1	[1]	SAMN08574914	H	84	38
T3Sed10_310R2	[1]	SAMN08574959	H	82	38
T3Sed10_183	[1]	SAMN08574879	H	78	48
T3Sed10_356	[1]	SAMN08574985	H	74	-
2CL.003	[3]	*	H	73	-
T3Sed10_256R1	[1]	SAMN08574923	H	72	-
T3Sed10_340	[1]	SAMN08574977	H	72	-
T3Sed10_209R1	[1]	SAMN08574895	H	70	-
T3Sed10_224	[1]	SAMN08574906	H	69	-
T3Sed10_187	[1]	SAMN08574881	H	66	-
CSSed10_255	[1]	SAMN08574516	H	64	-
T3Sed10_319	[1]	SAMN08574963	H	61	-
T3Sed10_256R2	[1]	SAMN08574924	H	61	-
T3Sed10_228m	[1]	SAMN08574908	H	61	-
T3Sed10_368	[1]	SAMN08574991	H	60	-
CSSed10_411	[1]	SAMN08574603	H	53	-
T3Sed10_210R1	[1]	SAMN08574897	H	50	-
CSSed10_487	[1]	SAMN08574631	H	46	-
