# Supplementary material for: Genome-Resolved Metagenomics Extends the Environmental Distribution of the Verrucomicrobia Phylum to the Deep Terrestrial Subsurface
Source: mSphere. 2019 Dec 18;4(6):e00613-19. doi: 10.1128/mSphere.00613-19 (PMC6920513; doi:10.1128/mSphere.00613-19)
Supplement: Supplementary file 4 [file mSphere.00613-19-st003.docx]

			OTU taxonomy (order I family I ID)														
**Sample ID**	**Shale gas well**	**Sample type**	Pedosphaerales I Pedosphaeraceae I FJ800525.1.1521	Methylacidiphilales I Methylacidiphilaceae I GU305773.1.1492	Opitutales I Opitutaceae I FR853407.1.1523	Verrucomicrobiales I Verrucomicrobiaceae I JN868733.1.1521	Opitutales I Puniceicoccaceae I HM856573.1.1433	Opitutales I Opitutaceae I EU801097.1.1446	Verrucomicrobiales I Verrucomicrobiaceae I JN869208.1.1538	Verrucomicrobiales I Verrucomicrobiaceae I FJ612430.1.1454	Opitutales I Puniceicoccaceae I 310811400 2b471d4161f24672a7221865	Opitutales I Puniceicoccaceae I b46cd94fa403d8ba9270ae03006ce888	Opitutales I Puniceicoccaceae I f8ba2ca05ce6d2b77c3e9aa8cbffa381	Opitutales I Opitutaceae I 741b6a215850019398b5096f3f90cf0f	Opitutales I Opitutaceae I 46b881a45ad6c9c71c6d64239c99dbcb	Verrucomicrobiales I Verrucomicrobiaceae I34c5e4dcc4d0658e7ed861262660 1064	Opitutales I Opitutaceae I 0f84c7121c5f75e20995ff40a1443c46
Marcellus_1_input	Marcellus-1	Input	0.0	0.0	0.0	0.0	0.0	0.0	0.0	0.0	0.0	0.0	0.0	0.0	0.0	0.0	0.0
Marcellus_1_T7	Marcellus-1	Flowback fluid (day 7)	0.0	0.0	0.0	0.0	0.0	0.0	0.0	0.0	0.0	0.0	0.0	0.0	0.0	0.0	0.0
Marcellus_1_T13	Marcellus-1	Flowback fluid (day 13)	0.0	0.0	0.0	0.0	0.0	0.0	0.0	0.0	0.0	0.0	0.0	0.0	0.0	0.0	0.0
Marcellus_1_T82	Marcellus-1	Produced water (day 82)	0.0	0.0	0.0	0.0	0.0	0.0	0.0	0.0	0.0	0.0	0.0	0.0	0.0	0.0	0.0
Marcellus_1_T328	Marcellus-1	Produced water (day 328)	0.0	0.0	0.0	0.0	0.0	0.0	0.0	0.0	0.0	0.0	0.0	0.0	0.0	0.0	0.0
Marcellus_4_T142	Marcellus-4	Produced water (day 142)	0.0	0.0	0.0	0.0	0.0	0.0	0.0	0.0	0.0	0.0	0.0	0.0	0.0	0.0	0.0
Marcellus_4_T303	Marcellus-4	Produced water (day 303)	0.0	0.0	0.0	0.0	0.0	0.0	0.0	0.0	0.0	0.0	0.0	0.0	0.0	0.0	0.0
W420140711FC	Utica-2	Input (chemicals)	0.3	0.0	0.0	0.0	0.0	0.0	0.0	0.0	0.0	0.0	0.0	0.0	0.0	0.0	0.0
W420140711HT	Utica-2	Input (holding tank)	0.4	1.1	0.0	0.0	0.0	0.0	0.0	0.0	0.0	0.0	0.0	0.0	0.0	0.0	0.0
W420140711LW	Utica-2	Input (lake water)	0.4	0.6	0.0	0.0	0.0	0.0	0.0	0.0	0.0	0.0	0.0	0.0	0.0	0.0	0.0
W420140716FC	Utica-2	Input (chemicals)	0.5	0.0	0.0	0.0	0.0	0.0	0.0	0.0	0.0	0.0	0.0	0.0	0.0	0.0	0.0
Utica_2_86	Utica-2	Produced water (day 86)	0.0	0.0	0.0	0.0	0.0	0.0	0.0	0.0	0.0	0.0	0.0	0.0	0.0	0.0	0.0
Utica_2_87	Utica-2	Produced water (day 87)	0.0	0.0	0.0	0.0	0.0	0.0	0.0	0.0	0.0	0.0	0.0	0.0	0.0	0.0	0.0
Utica_2_88	Utica-2	Produced water (day 88)	0.0	0.0	0.0	0.0	0.0	0.0	0.0	0.0	0.0	0.0	0.0	0.0	0.0	0.0	0.0
Utica_2_92	Utica-2	Produced water (day 92)	0.0	0.0	0.0	0.0	0.0	0.0	0.0	0.0	0.0	0.0	0.0	0.0	0.0	0.0	0.0
Utica_2_94	Utica-2	Produced water (day 94)	0.0	0.0	0.0	0.0	0.0	0.0	0.0	0.0	0.0	0.0	0.0	0.0	0.0	0.0	0.0
Utica_2_96	Utica-2	Produced water (day 96)	0.0	0.0	0.0	0.0	0.0	0.0	0.0	0.0	0.0	0.0	0.0	0.0	0.0	0.0	0.0
Utica_2_98	Utica-2	Produced water (day 98)	0.0	0.0	0.0	0.0	0.0	0.0	0.0	0.0	0.0	0.0	0.0	0.0	0.0	0.0	0.0
Utica_2_105	Utica-2	Produced water (day 105)	0.0	0.0	0.0	0.0	0.0	0.0	0.0	0.0	0.0	0.0	0.0	0.0	0.0	0.0	0.0
Utica_2_112	Utica-2	Produced water (day 112)	0.0	0.0	0.0	0.0	0.0	0.0	0.0	0.0	0.0	0.0	0.0	0.0	0.0	0.0	0.0
Utica_2_126	Utica-2	Produced water (day 126)	0.0	0.0	0.0	0.0	0.0	0.0	0.0	0.0	0.0	0.0	0.0	0.0	0.0	0.0	0.0
Utica_2_140	Utica-2	Produced water (day 140)	0.0	0.0	0.0	0.0	0.0	0.0	0.0	0.0	0.0	0.0	0.0	0.0	0.0	0.0	0.0
Utica_2_154	Utica-2	Produced water (day 154)	0.0	0.0	0.0	0.0	0.0	0.0	0.0	0.0	0.0	0.0	0.0	0.0	0.0	0.0	0.0
Utica_2_175	Utica-2	Produced water (day 175)	0.0	0.0	0.0	0.0	0.0	0.0	0.0	0.0	0.0	0.0	0.0	0.0	0.0	0.0	0.0
Utica_2_204	Utica-2	Produced water (day 204)	0.0	0.0	0.0	0.0	0.0	0.0	0.0	0.0	0.0	0.0	0.0	0.0	0.0	0.0	0.0
Utica_2_302	Utica-2	Produced water (day 302)	0.0	0.0	0.0	0.0	0.0	0.0	0.0	0.0	0.0	0.0	0.0	0.0	0.0	0.0	0.0
Utica_3_input2_FT	Utica-3	Input	0.0	0.0	0.0	3.6	0.6	0.3	0.1	0.1	0.0	0.0	0.0	0.0	0.0	0.0	0.0
Utica_3_input1_RT	Utica-3	Input	0.0	0.0	0.0	0.0	0.0	0.0	0.0	0.0	0.0	0.0	0.0	0.0	0.0	0.0	0.0
S_input_2_FT	Utica-3	Input	0.0	0.0	0.0	0.0	0.0	0.0	0.0	0.0	0.0	1.9	0.0	1.3	0.0	0.0	0.0
S_input_3_FT	Utica-3	Input	0.0	0.0	0.0	0.0	0.0	0.0	0.0	0.0	2.5	0.0	1.6	0.0	1.3	0.7	0.5
Utica_3_T1	Utica-3	Flowback fluid (day 1)	0.0	0.0	0.0	0.0	0.0	0.0	0.0	0.0	0.0	0.0	0.0	0.0	0.0	0.0	0.0
Utica_3_T9	Utica-3	Flowback fluid (day 9)	0.0	0.0	0.0	0.0	0.0	0.0	0.0	0.0	0.0	0.0	0.0	0.0	0.0	0.0	0.0
Utica_3_T17	Utica-3	Flowback fluid (day 17)	0.0	0.0	0.6	0.0	0.0	0.0	0.0	0.0	0.0	0.0	0.0	0.0	0.0	0.0	0.0
Utica_3_T23	Utica-3	Flowback fluid (day 23)	0.0	0.0	0.0	0.0	0.0	0.0	0.0	0.0	0.0	0.0	0.0	0.0	0.0	0.0	0.0
Utica_3_T59	Utica-3	Produced water (day 59)	0.0	0.0	0.0	0.0	0.0	0.0	0.0	0.0	0.0	0.0	0.0	0.0	0.0	0.0	0.0
[illegible]	[illegible]	[illegible]	[illegible]	[illegible]	[illegible]	[illegible]	[illegible]	[illegible]	[illegible]	[illegible]	[illegible]	[illegible]	[illegible]	[illegible]	[illegible]	[illegible]	[illegible]
