# Supplementary material for: Genome-Resolved Metagenomics Extends the Environmental Distribution of the Verrucomicrobia Phylum to the Deep Terrestrial Subsurface
Source: mSphere. 2019 Dec 18;4(6):e00613-19. doi: 10.1128/mSphere.00613-19 (PMC6920513; doi:10.1128/mSphere.00613-19)
Supplement: Supplementary file 5 [file mSphere.00613-19-st004.docx]

Function name	Kegg Number	E.C. Number	Gene
2-component system OmpR family sensor histidine kinase	K07646	EC:2.7.13.3	kdpD
2-component system OmpR family sensor histidine kinase	K07667	EC:2.7.13.3	kdpE
K+transporting ATPase ATPase F chain	K01545	EC:7.2.2.6	kdpF
K+transporting ATPase ATPase A chain	K01546	EC:7.2.2.6	kdpA
K+transporting ATPase ATPase B chain	K01547	EC:7.2.2.6	kdpB
K+transporting ATPase ATPase C chain	K01548	EC:7.2.2.6	kdpC
Na+:H+ antiporter	K03315		nhaC
multicomponent Na+:H+ antiporter subunit A	K05565		mnhA
multicomponent Na+:H+ antiporter subunit B	K05566		mnhB
multicomponent Na+:H+ antiporter subunit C	K05567		mnhC
multicomponent Na+:H+ antiporter subunit D	K05568		mnhD
multicomponent Na+:H+ antiporter subunit E	K05569		mnhE
multicomponent Na+:H+ antiporter subunit F	K05570		mnhF
multicomponent Na+:H+ antiporter subunit G	K05571		mnhG
trkA type sodium hydrogen exchanger	K03499		trkA, ktrA
trkH type K uptake	K03498		trkH, ktrB
choline dehydrogenase	K00108	EC:1.1.99.1	betA
NAD/NADP-dependent betaine aldehyde dehydrogenase	K00130	EC:1.2.1.8	betB
glutamate 5-kinase	K00931	EC:2.7.2.11	proB
gamma-glutamyl phosphate reductase	K00147	EC:1.2.1.41	proA
pyrroline-5-carboxylate reductase	K00286	EC:1.5.1.2	proC
proline/betaine transporter	K03762		proP
proline/betaine (ProP effector)	K03607		proQ
glycine betaine/proline transport system ATP binding protein	K02000	EC:3.6.3.32	proV
glycine betaine/proline betaine transport system permease protein	K02001		proW
glycine betaine/proline betaine binding periplasmic protein	K02002		proX
glycine/sarcosine N-methyltransferase	K18896	EC:2.1.1.156	bsmA
sarcosine/dimethylglycine N-methyltransferase	K18897	EC:2.1.1.157	SDMT
betaine reductase complex component B subunit alpha	K21578	EC:1.21.4.4	grdI
maltose phosphorylase	K00691	EC:2.4.1.8	mdxK
glycerol transport system substrate binding protein	K17321		glpV
glycerol-3-phosphate responsive antiterminator	K17322		glpP
glycerophosphoryl diester phosphodiesterase	K17323		glpQ
glycerol transport system ATP binding protein	K17324		glpS
glycerol-3-phosphate transporter	K17325		glpT
sn-glycerol-3-phosphate ABC transporter	K05814		ugpA
sn-glycerol-3-phosphate import ATP binding protein	K05816		ugpC
sn-glycerol 3-phosphate transport system permease protein	K05815		ugpE
ABC transporter, substrate binding protein (Trehalose/maltose)	K10236		thuE
trehalose synthase/amylase	K05343		treS
trehalose-6-phosphate synthase	K00697	EC:2.4.1.15	ostA
trehalose-6-phosphate phosphatase	K01087	EC:3.1.3.12	ostB
trehalose/maltose import ATP binding protein	K10112	EC:7.5.2.1	malK
periplasmic trehalase	K01194	EC:3.2.1.28	treA
mannitol-1-phosphate 5-dehydrogenase	K00009	EC:1.1.1.17	mltD
1-pyrroline-5-carboxylate-dehydrogenase 2	K00294	EC:1.2.1.88	putC
PTS system trehalose-specific subunit EIIBC	K02818, K02819		treB
