# Supplementary material for: Genome-Resolved Metagenomics Extends the Environmental Distribution of the Verrucomicrobia Phylum to the Deep Terrestrial Subsurface
Source: mSphere. 2019 Dec 18;4(6):e00613-19. doi: 10.1128/mSphere.00613-19 (PMC6920513; doi:10.1128/mSphere.00613-19)
Supplement: Supplementary file 6 [file mSphere.00613-19-st005.docx]

Genome name	single copy gene		Genome accession	
	completeness %	overages %	NCBI	JGI IMG
***Candidatus* Marcellius**	*100*	0	SAMN10737522	
Akkermansia muciniphila ATCC BAA-835	100	0	SAMN00138213	
Coraliomargarita akajimensis DSM 45221	100	0	SAMN02598501	
Methylacidiphilum fumariolicum SolV	100	0	SAMEA2682029	
Methylacidiphilum infernorum V4	100	0	SAMN02604183	
Opititae-40	100	0		Ga0081625
Opitutaceae bacterium (Lacunisphaera limnophila) IG16b	100	0	SAMN04880612	Ga0176001
Opitutaceae bacterium TAV1	100	0	SAMN02261400	Ga0024908
Opitutaceae bacterium TAV5	100	0	SAMN02261410	Ga0025066
Opitutus terrae PB90-1	100	0	SAMN00000285	
TE1301	100	0		Ga0048032
Verrucomicrobia bacterium GWF2_51_19	100	0		Ga0154578
Verrucomicrobia bacterium IMCC26134	100	0	SAMN03452270	Ga0077870
Verrucomicrobium sp. 3C	100	0	SAMN02256457	
Verrucomicrobium sp. BvORR034	100	0	SAMD00036767	Ga0056855
Verrucomicrobium sp. BvORR106	100	0	SAMD00036768	Ga0056857
Verrucomicrobium spinosum DSM 4136	100	0	SAMN02436175	
Pedos_66_17	100	3	SAMN10737521	
Candidatus Xiphinematobacter sp. Idaho Grape	97	0	SAMN04001717	Ga0175490
Kiritimatiella glycovorans strain L21-Fru-AB	97	0	SAMN03329217	
Terrimicrobium sacchariphilum NM-5	97	0	SAMD00048637	
Candidatus Udaeobacter copiosus	94	3		Ga0111076
ME12612	94	0		Ga0047939
TE1800	94	0		Ga0059290
TE4605	94	0		Ga0048054
Verrucomicrobia_bacterium_RIFCSPLOWO2_12_FULL_64_8	94	10		Ga0156277
ME12657	90	0		Ga0047940
TH2519	90	0		Ga0059282
TH4590	90	0		Ga0048105
Verrucomicrobia bacterium RIFCSPHIGHO2_12_FULL_41_10	90	3		Ga0155638
TH3004	87	0		Ga0048085
ME30509	84	0		Ga0047975
TH2746	84	0		Ga0048081
TH2747	84	0		Ga0048082
Verrucomicrobia bacterium GWC2_42_7	84	3		Ga0154466
Verrucomicrobia bacterium GWF2_62_7	77	0		Ga0154606
ME3880	71	0		Ga0047989
Verrucomicrobia bacterium LP2A	71	0	SAMN02584925	
TH4820	68	0		Ga0048108
Verrucomicrobia bacterium CG1_02_43_26	68	0	SAMN04328238	
ME6381	65	0		Ga0048009
ME8366	65	0		Ga0048023
TH4093	58	0		Ga0048099
Verrucomicrobia BACL9 MAG-120507-bin52	55	0	SAMN03741975	
Chthoniobacter flavus Ellin428	52	0	SAMN00000678	
ME12173	45	0		Ga0047937
ME2014	45	0		Ga0047962
Opitutaceae bacterium TSB47	30	0	SAMN04441843	Gp0156911
Rubritalea squalenifaciens DSM 18772	25	0	SAMN02745181	Gp0013232
Diplosphaera colotermitum (Geminisphaera colitermitum) TAV2	19	0	SAMN02256391	Gp0000926
Methylacidiphilum_kamchatkense_Kam1	10	0	SAMN02996356	Ga0070832
Verrucomicrobia_bacterium_BACL9_MAG-120820-bin42	10	0	SAMN03742025	
Prosthecobacter debontii ATCC 700200	3	0	SAMN02745166	
Rubritalea marina DSM 17716	3	0	SAMN02441342	Gp0013231
Verrucomicrobia_bacterium_13_1_20CM_4_55_9	3	0	SAMN04327797	
Coraliomargarita sp CAG312	0	0	SAMEA3138785	
Haloferula_sp._BvORR071	0	0	SAMD00036766	Ga0056856
Pedosphaera parvula Ellin514	0	0	SAMN00000631	
Verrucomicrobia bacterium_13_1_20CM_3_54_17	0	0	SAMN04327759	
Verrucomicrobia bacterium 13_1_20CM_4_54_11	0	0	SAMN04327788	
Verrucomicrobia bacterium 13_1_20CM_54_28	0	0	SAMN04327675	
Verrucomicrobia bacterium 13_1_40CM_4_54_4	0	0	SAMN04327898	
Verrucomicrobia bacterium Ga0077533	0	0	SAMN04254501	
Verrucomicrobiales bacterium NORP190	0	0	SAMN07569004	
